# Supplementary material for: Genome-Wide Identification and Expression Analysis of Growth-Regulating Factors in Eucommia ulmoides Oliver (Du-Zhong)
Source: Plants (Basel). 2024 Apr 24;13(9):1185. doi: 10.3390/plants13091185 (PMC11085888; doi:10.3390/plants13091185)
Supplement: Supplementary file 1 [file plants-13-01185-s001.zip › Supplementary Figures.pdf]

Supplementary Data S1. The information of candidate EuGRFs identified in *E. ulmoides* through BLAST and HMM search.
Supplementary Data S2. The information of 38 candidate eul-miR396s identified in *E. ulmoides*.
Supplementary Data S3. The information of linked pairs of between *EuGRFs* with *GRFs* in *A. thaliana* and *E. ulmoides*.
Supplementary Data S4. The information of cis-elements on promoter regions of 8 *EuGRFs*.
Supplementary Data S5. Query Sequences of miRNA396s used for Local BLAST search.
Supplementary Data S6. The list of primer sequences used in this paper.
Supplementary Data S7. The GRFs IDs and plant species used in the Maximum Likelihood tree.
Supplementary Data S8. The amino acids sequences of EuGRFs identified in our unpublished genome.
Supplementary Data S9. The log file of the Maximum Likelihood tree constructed in this paper.
Supplementary Data S10. The collinearity file within the *E. ulmoides* genome.

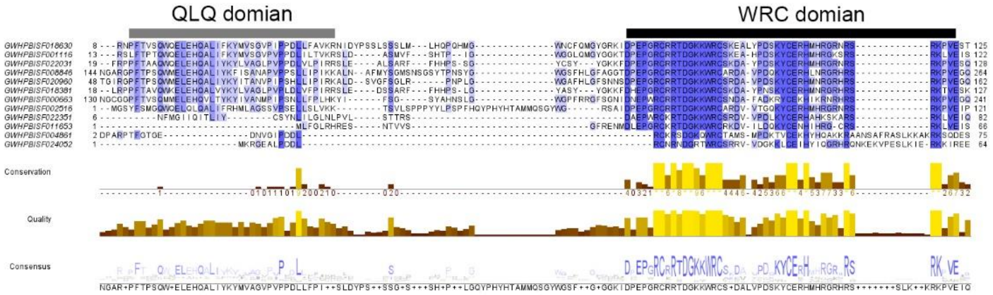

Figure S1. The amino acids sequence alignment of 12 GRF candidate proteins using MAFFT with default settings.

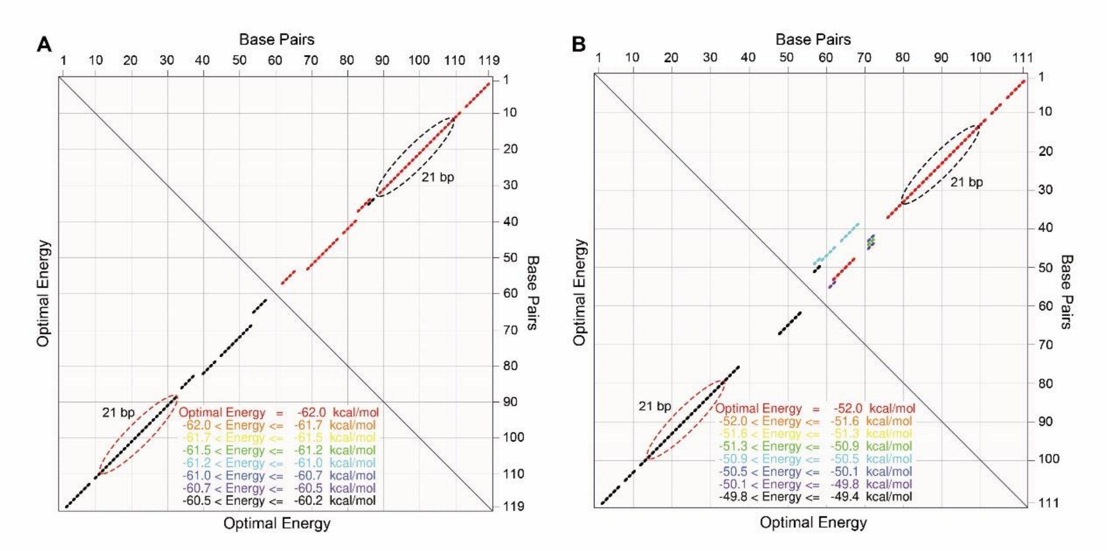

Figure S2. The energy dot plot for the hairpin structure formed by pre-miR396a (A) and pre-miR396b (B). The computed folding of pre-miR396a contains 51 base pairs out of 53 (96.2%) in the energy dot plot. The computed folding of pre-miR396b contain 50 base pairs out of 61 (82.0%) in the energy dot plot. The dashed lines represent the nucleotide bases of mature

eul-miR396s.

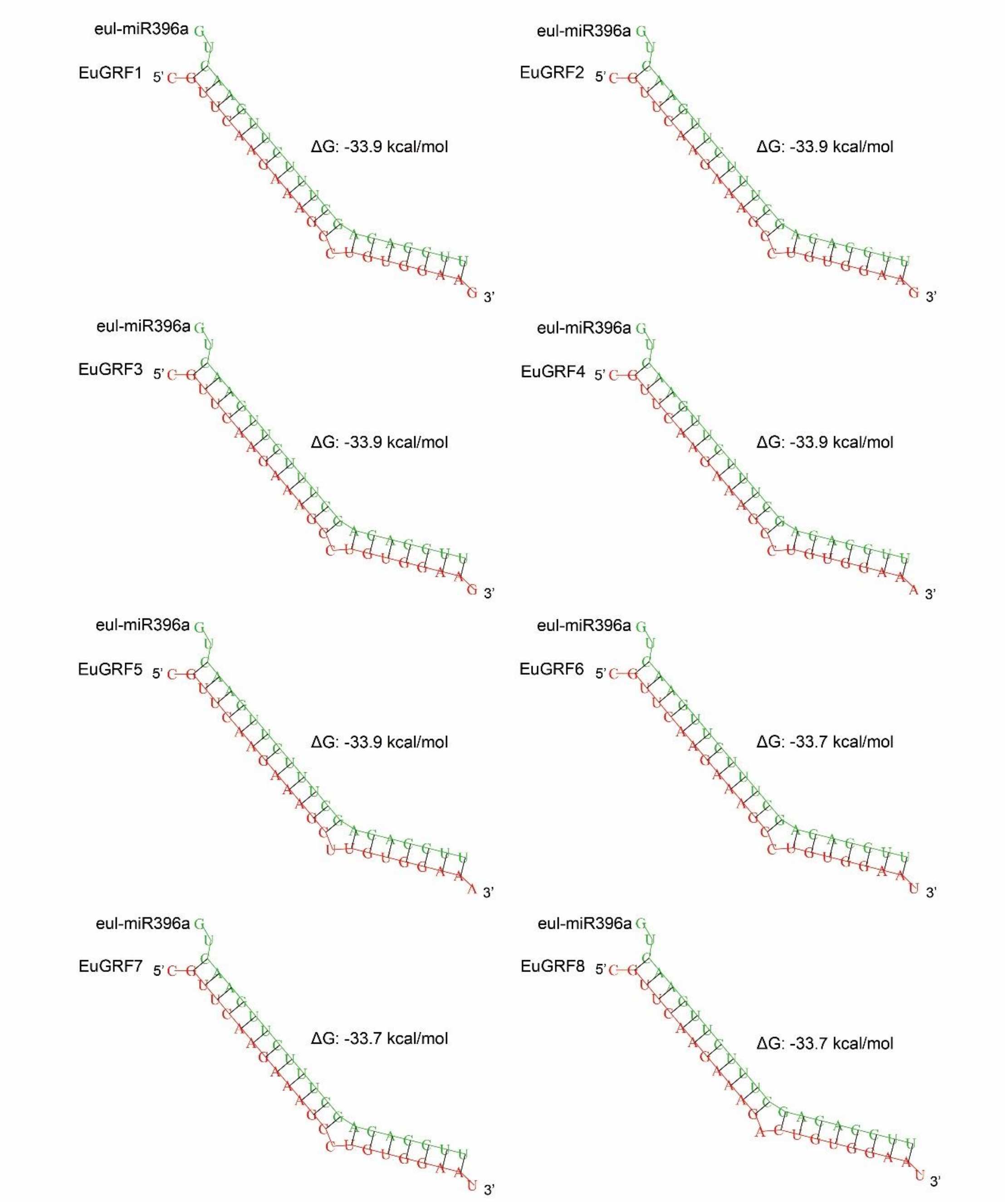

Figure S3. The degree of complementarity between eul-miR396a and all EuGRFs. The free energies of duplex structures were displayed on the right.

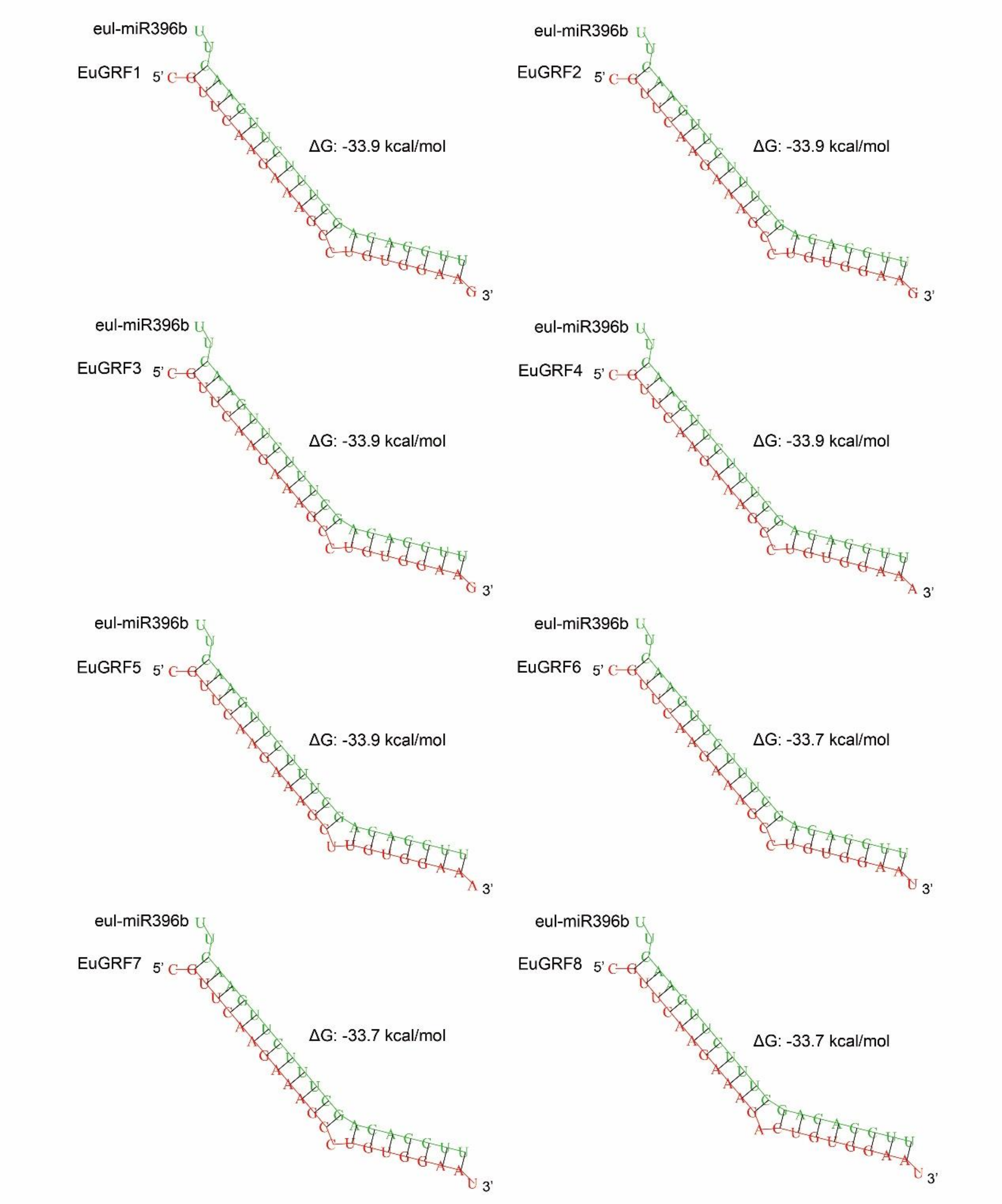

Figure S4. The degree of complementarity between eul-miR396b and all EuGRFs. The free energies of duplex structures were displayed on the right.

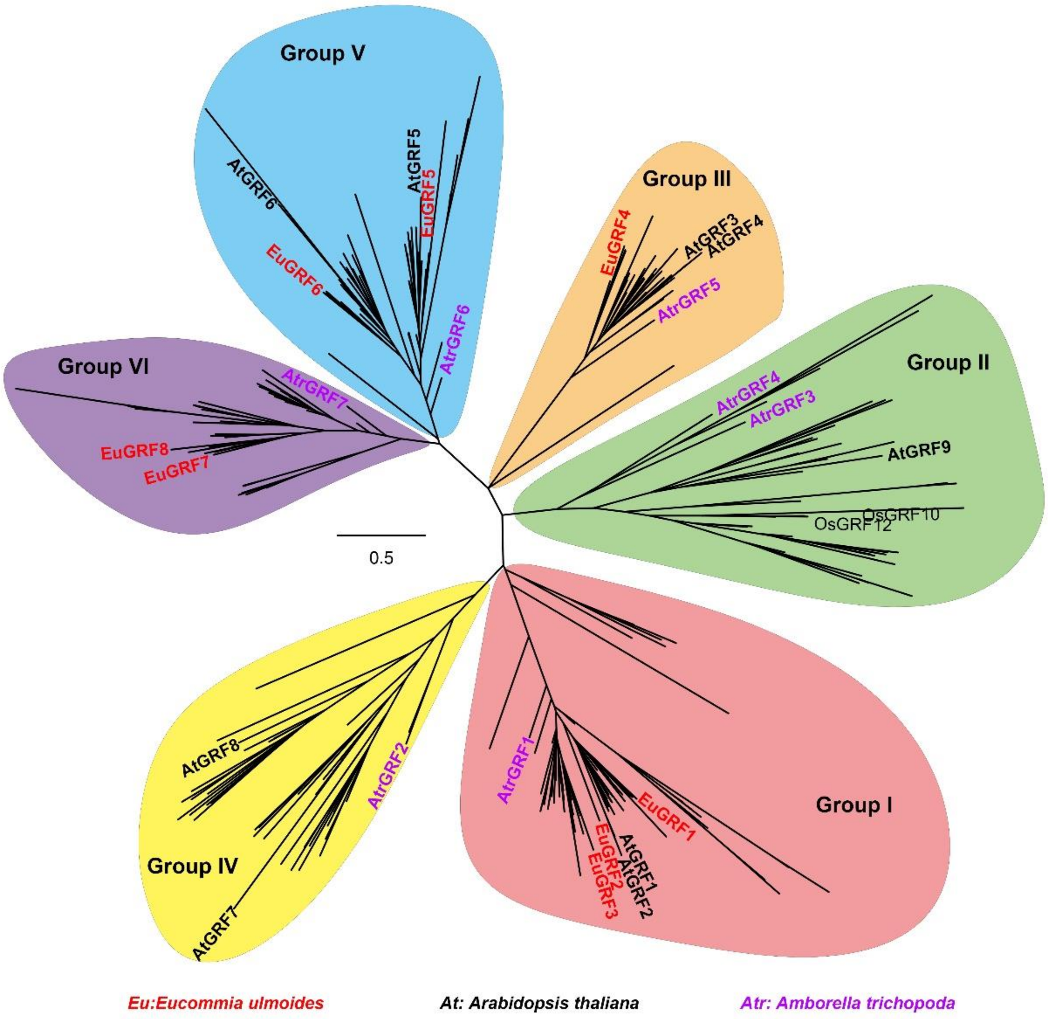

Figure S5. The Maximum Likelihood tree constructed using protein sequences of 290 GRFs derived from 27 terrestrial species. The scale bar indicates the branch length.

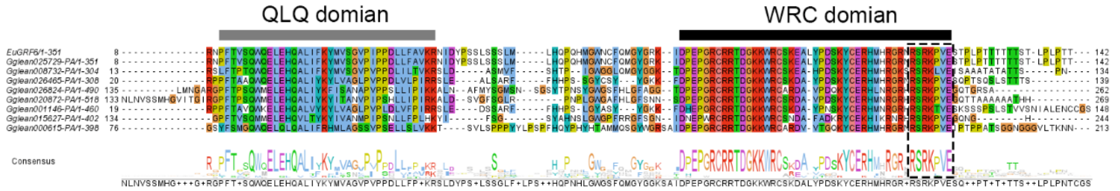

Figure S6. The amino acids sequence alignment of EuGRFs identified in our unpublished genome. The sequence alignment was performed using MAFFT with default settings. The conserved "RSRK-VE" motif is depicted within the dashed enclosure.
